# Assessment of SGLT2 inhibitors' safety and discontinuation causes in patients with advanced chronic kidney disease: insights from a real-world data analysis

**DOI:** 10.1093/ckj/sfae169

**Published:** 2024-07-03

**Authors:** Peggy Perrin, Clotilde Muller, Yves Dimitrov, Francois Chantrel, Marie Heitz, Amandine Woerly, Dorothée Bazin, Anne-Laure Faller, Thierry Krummel, Thierry Hannedouche

**Affiliations:** Department of Nephrology, Dialysis and Transplantation, Strasbourg University Hospital, Strasbourg, France; INSERM UMS S1109, LabEx Transplantex, Fédération de Médecine Translationnelle de Strasbourg (FMTS), Strasbourg University Hospital, Strasbourg, France; Department of Nephrology Groupe Hospitalier Saint-Vincent, Clinique Ste-Anne, Service de Néphrologie, Strasbourg, France; Renal Research Division, AURAL Strasbourg, Strasbourg, France; Renal Research Division, AURAL Strasbourg, Strasbourg, France; Department of Nephrology, CH Haguenau, Haguenau, France; Renal Research Division, AURAL Strasbourg, Strasbourg, France; Department of Nephrology Groupe Hospitalier de la Région Mulhouse et Sud Alsace, Hôpital Emile Muller, Mulhouse, France; Department of Nephrology, Colmar Hospital, Colmar, France; Department of Nephrology, Dialysis and Transplantation, Strasbourg University Hospital, Strasbourg, France; Department of Nephrology, Dialysis and Transplantation, Strasbourg University Hospital, Strasbourg, France; Department of Nephrology Groupe Hospitalier Saint-Vincent, Clinique Ste-Anne, Service de Néphrologie, Strasbourg, France; Renal Research Division, AURAL Strasbourg, Strasbourg, France; Department of Nephrology, Dialysis and Transplantation, Strasbourg University Hospital, Strasbourg, France; Department of Nephrology, Dialysis and Transplantation, Strasbourg University Hospital, Strasbourg, France; Renal Research Division, AURAL Strasbourg, Strasbourg, France

To the Editor,

Recent randomized trials [1, 2] have demonstrated the renal and cardiovascular benefit of sodium-glucose cotransporter 2 inhibitors (SGLT2i) in chronic kidney disease (CKD) patients, irrespective of diabetes status. However, real-world data on their use in advanced CKD stages remain scarce. This study aims to assess the safety and discontinuation reasons of SGLT2i in this demographic.

We conducted a retrospective analysis of 271 patients with stage 4 and non-dialysed stage 5 CKD, from January 2020 to December 2022, across five nephrology units (CKD-REAL study). The median initiation of SGLT2i was 356 days post-stage 4 diagnosis, predominantly with dapagliflozin (266 patients) and empagliflozin (six patients). Safety profiles and eGFR trajectories were monitored at 6 and 12 months, with a minimum follow-up of 6 months.

At SGLT2i initiation, the mean age was 71 ± 11 years, with diabetes prevalence at 81%. The aetiologies included diabetic nephropathy (39%), vascular nephropathy (28%), and glomerulonephritis (14%). The mean GFR was 27.2 ± 4.9 ml/min/1.73 m^2^, and median urinary albumin-to-creatinine (ACR) was 200 mg/g. Heart failure was present in 31.4%, while 61.6% had cardiovascular disease. Most were on renin-angiotensin system (RAS) inhibitors (81.5%) and diuretics (72.6%) (Table 1).

**Table 1:** Characteristics of patients at initiation of SGLT2 inhibitor and safety outcomes 6 and 12 months after initiation.

	
	***n* = 271**
**Characteristic at initiation of SGLT2i**	
Age (year)	71.1 ± 10.9
Female sex (%)	81 (29.9)
Type 2 diabetes, *n* (%)	213 (78.6)
Type 1 diabetes, *n* (%)	6 (2.2)
Diabetic nephropathy, *n* (%)	105 (38.8)
Vascular nephropathy, *n* (%)	75 (27.7)
Interstitial nephropathy, *n* (%)	10 (3.7)
Other glomerulopathy, *n* (%)	39 (14.4)
Undetermined/other, *n* (%)	42 (15.5)
History of heart failure, *n* (%)	85 (31.4)
Cardiovascular disease ^a^, *n* (%)	167 (61.6)
Body-mass index (kg/m^2^)	31.0 (6.5)
Systolic Blood pressure (mmHg)	136.8 (18.8)
Diastolic Blood pressure (mmHg)	74.9 ± 11.9
eGFR (ml/min/1.73 m^2^)	27.2 ± 4.9
Distribution, *n* (%)	
eGFR 30 to <45 ml/min/1.73 m^2^	63 (23.3)
eGFR 15 to <30 ml/min/1.73 m^2^	204 (75.3)
eGFR 25 to <30 ml/min/1.73 m^2^	141 (52.0)
eGFR 15 to <25 ml/min/1.73 m^2^	63 (23.3)
eGFR <15 ml/min/1.73 m^2^	4 (1.5)
Median UACR, mg/g (IQR1, IQR3), *n* = 185	200 (51, 699)
UACR >30 mg/g (%), *n* = 185	147 (79.5%)
hbA1c, *n* = 210	7.1 1.2
**Baseline medication**	
ACE inhibitor or ARB, *n* (%)	220 (81.5)
Diuretic, *n* (%)	196 (72.6)
Insulin, *n* (%)	122 (45.2)
GLP-1, *n* (%)	53 (19.6)
Metformin, *n* (%)	33 (12.2)
Gliptin, n (%)	50 (18.5)
Sulfonylurea, *n* (%)	13 (4.8)
Number of antihypertensive classes	2.9 (1.2)
**Safety outcomes**	
Discontinuation at M6, *n* (%)	28 of 271 (10.3%)
Discontinuation at M6 for SAE, *n* (%)	2 of 271 (0.7%)
Subjects with any SAE between D0-M6, *n*^b^	41 of 271 (15.1%)
Discontinuation between M6 and M12, *n* (%)	11 of 154 (7.1%)
Discontinuation between M6 and M12 for SAE, *n* (%)	3 of 154 (1.9%)
Causes of discontinuation at M6 or at M12, *n* = 39^c^	
GFR decline or AKI	27 (69.2%)
Urinary or genital infection	4 (10.3%)
Digestive disorders	3 (7.7%)
Lipothymia	2 (5.1%)
Other or not defined	4 (10.2%)
Non-observance	2 (5.1%)

Data are shown as mean ± SD or median (Q1–Q3). ACE denotes angiotensin-converting enzyme; AKI, acute kidney disease; ARB, angiotensin-receptor blocker; eGFR, estimated glomerular filtration rate; SAE, serious adverse event; and UACR, urinary albumin-to-creatinine ratio. eGFR was calculated using CKD-EPI equation. UACR was calculated with albumin in milligrams and creatinine in grams.

a Cardiovascular disease was defined as a history of coronaropathy, peripheral artery disease, heart failure, ischaemic stroke, transient ischaemic attack, haemorrhagic stroke. Transient discontinuations are not indicated in this table.

b SAE were censored if occurred after discontinuation.

^c^The total causes exceed 100% because several associated reasons for discontinuation may occur.

At 6 months, 28 patients (10.3%) had discontinued SGLT2i use, and by 12 months, on additional 7.1% had stopped (flowchart, figure S1, Table S1). The primary reason for discontinuation was eGFR decline, accounting for 27 (69.2%) cases, including hospitalization for five patients. During this period, 54 serious adverse events (SAEs) occurred among 41 patients (15.1%), with a 3.7% incidence of events potentially attributable to gliflozin, such as acute kidney injury (AKI) and urinary tract infection (Tables S3, S4).

Interestingly, patients who discontinued SGLT2i at 6 months experienced a significantly steeper decline in eGFR compared to those who continued treatment (−7.3 ± 4.6 ml/min/1.73 m^2^ vs −3.8 ± 4.5, *P *< .001, Fig. 1a). Multivariate analysis revealed that discontinuation at month 6 was independently correlated with lower initial GFR (AHR 0.88, 95CI 0.80–0.96), a greater drop in eGFR (AHR = 0.81, 95%CI 0.72–0.90), and cardiac failure (AHR 4.24, 95 CI 1.78–10.10) (Table S2).

**Figure 1:**
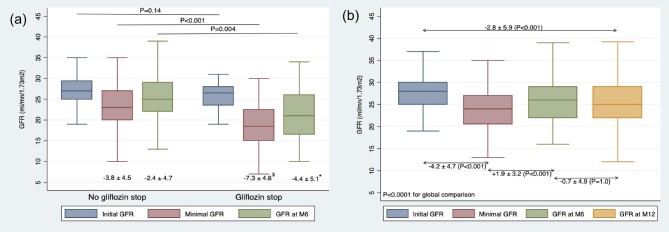
Changes in eGFR in (a) patients on SGLT2i and those with discontinuation at month 6 and in (b) patients on SLT2i at M12. Minimal GFR represents the lowest recorded GFR during the initial 6 months. Comparison for GFR change: $*P *< .001; *P = .032. In (b), linear mixed models confirmed the ANOVA results with a coefficient of −.52 (*P* < .001).

At 6 months, patients continuing SGLT2i showed significant reductions in weight, ACR, blood pressure, and a reduce reliance on diuretics and RAS inhibitors (Table S1). For the 143 patients on SGLT2i at 12 months, the eGFR slope was −2.8 ± 5.9 ml/min/1.73 m^2^ (Fig. 1b).

This investigation stands as the first real-world examination of SGLT2i in advanced CKD patients. Compared with stage 4 CKD participants from the DAPA-CKD trial [3], our real-world cohort was generally older, had a higher diabetes prevalence, a doubled incidence of cardiovascular disease, more frequent diuretic use, and lower baseline albuminuria. Additionally, vascular nephropathy was more common [1].

The findings confirm a reassuring safety profile for SGLT2i in advanced CKD patients, consistent with trial data. However, the discontinuation rate was significant, at ∼10% by 6 months, and an additional 7% between 6 and 12 months. The primary discontinuation driver was a notable GFR decline. The DAPA-CKD trial reported a median 2.4-year follow-up discontinuation due to adverse events at ∼4.8% for CKD stage 3 and 10% for stage 4, comparable to both the treatment and placebo groups [2]. A *post hoc* analysis suggested that patients who experienced an initial eGFR reduction of >10% following dapagliflozin initiation exhibited a slower long-term eGFR decline [4], proposing that a modest initial eGFR decrease should not trigger therapy discontinuation.

Our study identified an independent link between therapy cessation at month 6 with lower initial GFR, a history of cardiac failure, and a significant GFR drop. Moreover, GFR decline also led to temporary therapy suspension in 3.3% of patients, who later resumed treatment. Withdrawal due to GFR decrease was connected with various conditions, including hospitalization in 18.5% of cases.

Despite its valuable insights, this study has limitations inherent to retrospective analyses. The real-world follow-up had less frequent visits and GFR assessments. The minimum GFR recorded in the first 6 months may not have distinguished between an initial GFR drop post-SGLT2i start and/or AKI episodes. Furthermore, documentation of the physician responsible for discontinuation was inconsistent, but remarkably, nephrologists often reintroduced treatment during follow-up, evidenced by the 8% of patients with temporary discontinuation within the first 6 months.

In conclusion, in a real-world setting with advanced CKD, discontinuation rates reached ∼10% at 6 months, surpassing those in trial contexts. The decline in GFR was the principal cause of discontinuation, observed in two-thirds of the instances. Nephrologists play a pivotal role in discerning the underlying causes of GFR decline, optimizing concomitant medications such as RAS inhibitors and diuretics, and considering therapy reintroduction, including in cases of heart failure where SGLT2i have demonstrated benefit, despite reduced eGFR.

## Supplementary Material

sfae169_Supplemental_File
