# Supplementary material for: Assessment of SGLT2 inhibitors' safety and discontinuation causes in patients with advanced chronic kidney disease: insights from a real-world data analysis
Source: Clin Kidney J. 2024 Jul 3;17(7):sfae169. doi: 10.1093/ckj/sfae169 (PMC11270012; doi:10.1093/ckj/sfae169)
Supplement: sfae169_Supplemental_File [file sfae169_supplemental_file.pdf]

Supplementary data

Assessment of SGLT2 Inhibitors' Safety and Discontinuation Causes in Patients with Advanced Chronic Kidney Disease: Insights from a Real-World Data Analysis

Index

Figure S1	Flowchart	p.2
Supplement	Patients and methods	p.3
Table S1	Characteristics and outcomes of patients on SGLT2i compared to patients with discontinuation at six months	p.4
Table S2	Multivariable analysis: factors associated with SGLT2 inhibitor discontinuing at month 6	p.5
Table S3	Causes of discontinuation and hospitalization, if required, in patients with iSGLT2 discontinuation (A) at M6 and in patients with transient discontinuation within the first six months, (B) at M12 and in patients with transient discontinuation between M6 and M12, and (C) in patients with discontinuation at M6 or at M12	p. 6,7
Table S4	Serious adverse events (SAE) and adverse events of interest in the first six months after SGLT2i initiation	p. 8

Figure S1: Flowchart

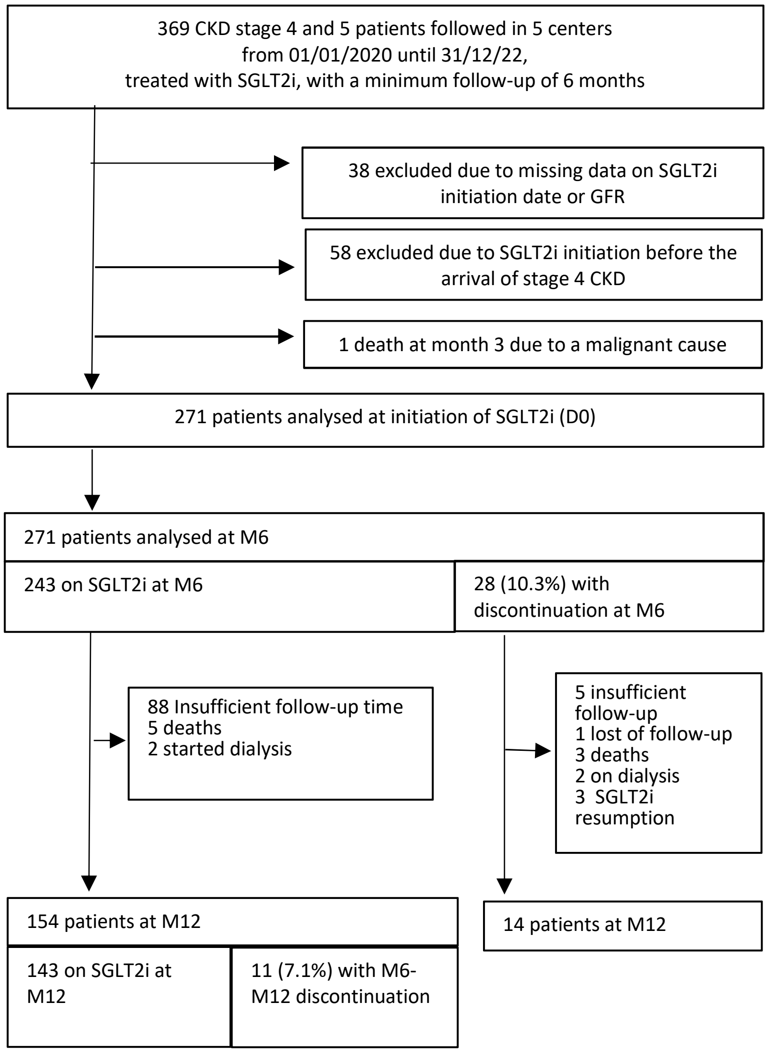

Supplement: Patients and methods

Patients

Patients in our study were enrolled in the 'forfait MRC' cohort within a clinical setting from January 1, 2020 until December 31, 2022, across five French centers (University Hospital of Strasbourg; Clinique saint-Anne, Strasbourg; Hospital of Haguenau, Hospital of Colmar, Hospital of Mulhouse). The 'forfait MRC' initiative reflective a pivotal reallocation of funds by French health authorities in December 2019 for managing stages 4 and 5 chronic kidney disease (CKD), excluding those undergoing dialysis. Eligibility for cohort inclusion required a diagnosis of stage 4 CKD, verified by an eGFR below 30 ml/min/1.73 m^2, documented twice over a three-month period. This prospective cohort mandated biannual submission of clinical data to the health authorities. Participants were retained in the cohort even if their eGFR fluctuated, provided that it dropped below the 30 ml/min/1.73 m^2 threshold at least once within a given year.

The CKD-REAL study is a retrospective evaluation of the 'forfait MRC' cohort members. For our analysis, we specifically included individuals who commenced treatment with SGLT2i more than six months prior. Those who had received kidney transplants were not considered for this study. The research protocol received ethical endorsement from the Strasbourg University Hospital Research Ethics Committee (CE-2022-9). The study procedures were in line with the Jardé law, ensuring that participants were duly informed about the data utilization and could exercise their right to dissent. This study is registered with clinicaltrials.gov (NCT05527626).

Analytical Approach

Quantitative data are reported as means ± standard deviations (SD) or medians with interquartile ranges, while categorical data are summarized as counts (n) and percentages (%). Given the non-normal distribution of our data ($p < 0.05$), non-parametric methodologies were applied for statistical analyses.

In differentiating the demographic, clinical, and therapeutic profiles of patients who discontinued or continued gliflozin after six months, the Mann-Whitney test was utilized for continuous variables, and Fischer's exact test was employed for categorical variables. We also applied the Wilcoxon matched-pairs signed-ranks test to compare parameters at the 12-month mark against baseline. Moreover, an analysis of variance (ANOVA) for repeated measures was conducted to explore GFR trends over the first year among those under treatment, and was checked with linear mixed models.

We further investigated potential discontinuation risk factors at the six-month interval that had shown a preliminary association ($p < 0.20$) in univariate analyses by incorporating them as covariates in a multivariable logistic regression framework (covariates included age, gender, history of cardiac failure, cardiovascular disease, baseline serum renal assay (BSRA), initial diuretic use, baseline GFR, and the maximal GFR variation within the initial six months).

All statistical evaluations were executed using Stata MP 14.1 (StataCorp, TX, USA), with bidirectional hypothesis testing, considering a p-value of less than 0.05 as indicative of statistical significance.

Table S1: Characteristics and outcomes of patients on SGLT2i compared to patients with discontinuation at six months

	Continuers N =243		Discontinuers n= 28		p
**Baseline**					
Age (year)	70.9 ± 11.2		72.5 ± 8.6		0.68
Female sex (%)	76 (31.3)		5 17.9		0.19
Diabetes (Type 2 or Type 1)	195 (80.2)		24 (85.7)		0.61
History of heart failure	67 (27.6)		18 (64.3)		<0.001
Cardio-vascular disease	145 (59.7)		22 (78.6)		0.064
Weight (kg)	89.4 ± 20.0		87.0 ± 16.7		0.55
Body mass index (kg/m^2)	31.1 ± 6.7		30.2 ± 5.1		0.47
Systolic blood pressure, mmHg	137.1 ± 18.8		134.0 ± 19.0		0.32
Diastolic blood pressure, mmHg	75.0 ± 11.6		74.0 ± 15.0		0.61
Nephropathy					0.58
Diabetic nephropathy (%)	94 (38.7)		11 (39.3)		
Vascular nephropathy (%)	69 (28.4)		6 (21.4)		
Other glomerulopathy (%)	37 (15.2)		2 (7.1)		
Interstitial nephropathy (%)	9 (3.7)		1 (3.6)		
Undetermined/other (%)	34 (14.0)		8 (28.6)		
eGFR ml/min/1.73 m^2	27.3 ± 5.0		25.9 ± 4.5		0.14
eGFR distribution:					0.578
30 to <45 ml/min/1.73 m^2	59 (24.8)		4 (14.3)		
15 to <30 ml/min/1.73 m^2	180 (74.1)		24 (85.7)		
< 15 ml/min/1.73 m^2	4 (1.7)		0		
UACR, mg/g	201 [46-690]		162 [61-1176]		0.56
HbA1c, g/dl	7.2 ± 1.2		7.0 ± 1.0		0.53
ACE inhibitor or ARB (%)	201 (83.1)		19 (67.9)		0.069
Diuretic (%)	172 (71.1)		24 (85.7)		0.12
Number of hypertensive drug classes	2.9 ± 1.2		2.8 ± 1.2		0.63
**Follow-up in the first 6 months**		Comparison to baseline values		Comparison to baseline values	
Minimal eGFR[a], ml/min/1.73 m^2	23.5 ± 5.5	<0.0001	18.6 ± 5.9	<0.0001	0.0001
Maximal change in eGFR, ml/min/1.73 m^2	-3.8 ± 4.5		-7.3 ± 4.6		0.0003
>10%	150 (61.7)		24 (85.7)		0.012
>30%	36 (14.8)		11 (39.3)		0.001
eGFR at M6, ml/min/1.73 m^2	25.3 ± 5.5	<0.0001	21.5 ± 6.3	0.0003	0.004
Change in eGFR at M6, ml/min/1.73 m^2	-2.4 ± 4.7		-4.4 ± 5.1		0.032
>10%	109 (44.9)		18 (64.3)		0.071
>30%	11 (4.5)		7 (25.0)		0.001
eGFR distribution at M6		<0.0001		0.120	0.008
<45 ml/min/1.73 m^2	46 (22.3)		3 (12.0)		
15 to <30 ml/min/1.73 m^2	153 (74.3)		17 (68.0)		
<15 ml/min/1.73 m^2	7 (3.4)		5 (20)		
M6 UACR, mg/g	165 [44-478]	0.031	160 [2-900]	0.88	0.84
Weight at M6 (kg)	88.3 ± 20.3	0.004	89.2 ± 15.9	0.94	0.76
Change in weight at M6 (kg)	-0.7 ± 3.7		-0.08 ± 5.5		0.66
hbA1c at M6	7.1 ± 1.0	0.58	6.9 ± 1.0	0.020	0.44
Systolic blood pressure at M6, mmHg	131.5 ± 16.6	<0.0001	134.3 ± 17.4	0.97	0.40
Diastolic blood pressure at M6, mmHg	73.0 ± 10.7	0.33	76.0 ± 12.4	0.78	0.15
ACE inhibitors or ARB at M6, mmHg (%)	200 ± 82.6	<0.001	16 ± 59.3	0.001	0.008
Diuretic at M6 (%)	168 ± 69.1	<0.001	24 ± 88.9	0.001	0.042
Number of antihypertensive classes at M6	2.8 ± 1.1	0.19	2.8 ± 1.1	0.99	0.85

a Minimal eGFR was the lower GFR reported in the 6 months

Data are shown as mean ± SD or median (Q1-Q3). ACE denotes angiotensin-converting enzyme, ARB angiotensin-receptor blocker, eGFR estimated glomerular filtration rate, and UACR urinary albumin-to-creatinine ratio. eGFR was calculated using CKD-EPI equation. UACR was calculated with albumin measured in milligrams and creatinine measured in grams. Cardiovascular disease was defined as a history of coronaropathy, peripheral artery disease, heart failure, ischemic stroke, transient ischemic attack, or hemorrhagic stroke

Table S2: Multivariable analysis: factors associated with SGLT2 inhibitor discontinuing at month 6

	OR	95CI	p
eGFR at initiation (per ml/min/1.73m^2)	0.876	0.797-0.963	0.006
Change in eGFR (per ml/min/1.73m^2)	0.809	0.727-0.901	<0.001
Cardiac failure	4.244	1.783-10.102	0.001
Female sex	0.40	0.14-1.18	0.10
Age (per year)	0.98	0.94-1.03	0.46
Cardiovascular disease	0.71	0.16-3.02	0.64
Diuretic at baseline	1.28	0.34-4.78	0.71
ACEi/ARB at baseline	0.73	0.26-2.02	0.54

Table S3: Causes of discontinuation and hospitalization, if required, in patients with iSGLT2 discontinuation (A) at M6 and in patients with transient discontinuation within the first six months, (B) at M12 and in patients with transient discontinuation between M6 and M12, and (C) in patients with discontinuation at M6 or at M12

A

**Subjects analyzed at M6, n=271**		Hospitalization, n
**Subjects with SGLT2i discontinuation at M6, n (%)**	**28 (10.3)**	**2 (0.7)**
**Causes - no.**		
GFR decline	20	2
GFR decline	14	0
GFR decline + dehydration	1	0
GFR decline + cardiac failure	1	1
GFR decline + liposarcoma recurrence	1	0
GFR decline (patient decision)	2	0
Acute kidney injury + diarrhea + pyelonephritis	1	1
Cutaneous intolerance	1	0
Digestive symptoms	2	0
Non-observance	2	0
Genital infection	1	0
Lipothymia (patient decision)	1	0
Not defined	1	0
**Subjects with transient discontinuation between D0 and M6, n (%)**	**22 (8.1)**	**7 (2.6)**
**Causes, n**		
GFR decline	9	3
GFR decline	5	1
Acute kidney injury + vomiting	1	1
GFR decline + urinary tract infection	2	1
GFR decline + coronary angiography	1	0
Asthenia	2	1
Asthenia + dyspnea	1	0
Cardiac events	2	2
Cholecystitis	1	1
Diabetes imbalance	1	0
Digestive symptoms (patient decision)	1	0
Urinary tract infection	1	0
Non-observance	2	0
Not defined	2	0

B

Subjects analyzed between M6 and M12, n=154		Hospitalization, n
**Subjects with discontinuation at M12, n (%)**	**11 (7.1)**	**3 (1.9)**
**Causes, n**		
GFR decline, total	7	3
GFR decline	4	0
GFR decline + cardiac failure	1	1
Acute kidney injury + urinary tract infection	1	1
Post-operative GFR decline + acute tubular necrosis	1	1
Urinary tract infection	1	0
Patient intolerance	1	0
Non-observance	1	0
Not defined	1	0
**Subjects with transient discontinuation between M6 and M12, n (%)**	**3 (1.9)**	**1 (0.6)**
**Causes , n**		
Urinary tract infection + cardiac failure	1	1
Diabetes imbalance	1	0
Out of stock	1	0

C

		Hospitalization, n
**Total subjects with discontinuation at M6 or M12**	**39**	**5**
**Causes, n**		
GFR decline, total	27	5
GFR decline	18	0
GFR decline + dehydration	1	0
GFR decline + cardiac failure	2	2
GFR decline + liposarcoma recurrence	1	0
GFR decline (patient decision)	2	0
Acute kidney injury + diarrhea + pyelonephritis	1	1
Acute kidney injury + urinary tract infection	1	1
Post-operative GFR decline + acute tubular necrosis	1	1
Cutaneous intolerance	1	0
Digestive symptoms	2	0
Non-observance	2	0
Genital infection	1	0
Lipothymia	2	0
Urinary tract infection	1	0
Patient intolerance	1	0
Not defined	2	0

Table S4: Serious adverse events (SAE) and adverse events of interest in the first six months after SGLT2i initiation

**Subjects with any SAE** (before discontinuation if appropriate)**, n (%)**	**41 of 271 (15.1)**
**SAE, number of events**	**54**
**Infections and infestations**	**6**
Cholangitis	1
Erysipelas	2
COVID	1
Viral bronchitis	1
Cholecystitis	1
**Cardiovascular disorders**	**20**
Angioplasty + stent	2
Coronary angiography	2
Cardiac failure	8
Angina	1
Carotid endarterectomy	2
Implantable Cardioverter Defibrillator	1
Myocardial infarction	1
Chest pain	1
Stent	1
Ischemic stroke	1
**Gastrointestinal disorders**	**2**
Diarrhea	1
Colectomy	1
**Metabolism and nutrition disorders**	**5**
Hyperkalemia	1
Metabolic acidosis	2
Diabetes imbalance	2
**Renal and urinary disorders**	**15**
Acute kidney injury	8
Hematuria	1
Urinary tract infection	4
Postoperative acute kidney injury	1
Renal biopsy	1
**Other disorders**	**6**
Vocal cords surgery	1
Asthenia	2
Rotator Cuff Repair	1
Hip replacement	2
**Subjects with Any SAE possibly related to gliflozine between D0 and M6**	**10**
Acute kidney injury+ Urinary tract infection	2
Acute kidney injury+ vomiting	1
Urinary tract infection + Hematuria	1
Urinary tract infection	1
Acute kidney injury+ cardiac failure	2
Acute kidney injury+ Metabolic acidosis	1
Acute kidney injury	2
**Subjects with Adverse Event of interest between D0 and M6**	
Death (malignant cause)	1
Dialysis or transplantation	0
CKD stage 5 (GFR<15 ml/min/1.73 m^2) non dialysis	8
Diabetic ketoacidosis	0
Urinary infection	7
Genital infection	1
Amputation	0
